# Three-dimensional (3D) tetra-culture brain on chip platform for organophosphate toxicity screening

**DOI:** 10.1038/s41598-018-20876-2

**Published:** 2018-02-12

**Authors:** Youngmi Koo, Brian T. Hawkins, Yeoheung Yun

**Affiliations:** 1 FIT BEST Laboratory, Department of Chemical, Biological, and Bio Engineering, North Carolina A&T State University, Greensboro, NC 27411 USA; 2 Discovery Science & Technology, RTI International, Research Triangle Park, Durham, NC 27709 USA; 3 WENeoBio Co., Gunsan-Si, Jeollabuk-do, 54001 South Korea; 4 Duke University Center for WaSH-AID, Durham, NC 27701 USA

## Abstract

Organophosphate-based compounds (OPs) represent a significant threat to warfighters (nerve agents) and civilian populations (pesticides). There is a pressing need to develop *in vitro* brain models that correlate to the *in vivo* brain to rapidly study OPs for neurotoxicity. Here we report on a microfluidic-based three-dimensional, four-cell tissue construct consisting of 1) a blood-brain barrier that has dynamic flow and membrane-free culture of the endothelial layer, and 2) an extracellular matrix (ECM)-embedded tissue construct with neuroblastoma, microglia, and astrocytes. We demonstrated this platform’s utility by measuring OP effects on barrier integrity, acetylcholinesterase (AChE) inhibition, viability and residual OP concentration with four model OPs. The results show that the OPs penetrate the blood brain barrier (BBB) and rapidly inhibit AChE activity, and that *in vitro* toxicity was correlated with available *in vivo* data. This paper demonstrates the potential utility of a membrane-free tetra-cultured brain on chip that can be scaled to high throughput as a cost-effective alternative method to animal testing.

## Introduction

Organophosphate-based compounds (OPs) pose an acutely toxic threat to both warfighters and civilian populations, creating an urgent need to develop better protective measures and therapeutic interventions^1^. OP poisoning causes the inhibition of acetylcholinesterase (AChE)^1–5^, leading to the accumulation of acetylcholine (ACh) in synapses and neuromuscular junctions and thus hyper-cholinergic activity that results in excitotoxicity, seizures, and brain damage^6–8^. Recent studies demonstrated that OPs generated oxidative stress and lipid peroxidation in different cell types and elevated the production of reactive oxygen species (ROS) and DNA damage^9,10^. Mitochondrial-mediated and caspase-regulated apoptosis are associated with different signaling mechanisms including the mitogen-activated protein kinase (MAPK) pathways and activation of proapoptotic markers-Bax, and caspases-9/3^11,12^. The blood brain barrier (BBB) provides the first line of defense for the central nervous system, and determines the rate at which OPs will reach the brain. The BBB is a selective diffusion barrier formed by specialized endothelial cells lining cerebral microvessels. These cells are characterized by high electrical resistance, epithelial-like tight junctions, and enriched expression of transport proteins that facilitate update of essential nutrients and efflux of xenobiotics^13^. The BBB interacts with other cellular components of the central nervous system (CNS), such as astrocytes, pericytes, and neuronal processes. However, many OPs readily cross the BBB, resulting in lethal CNS disruption^14^. Thus, understanding OP penetration of the BBB is the first critical piece of information required to predict acute neurotoxicity. This has been studied recently with the development of animal models of acute OP neurotoxicity^15–18^. However, relying exclusively on animal models severely hampers the speed with which new drug targets and candidate therapeutics can be identified and qualified, in addition to making development more expensive.

There is a clear need to develop more realistic *in vitro* brain models that simulate brain activities, mechanical environment, and complex physiological responses. Recent efforts to develop better *in vitro* brain models include brain organoids, mini-brain, microfluidic-based brain on a chip, tissue chip, and iPSC-derived microphysiological systems^19–21^. Recent studies account for differentiation to certain cellular subtypes of the human brain, including a myriad of neuronal subtypes, glial cells and endothelium. These *in vitro* systems may also be used to mimic diseases such as Alzheimer’s and Parkinson’s diseases^22^.

This paper reports on development of a perfusion-based3D brain culture platform that mimics the neurovascular environment. We constructed an *in vitro* brain model consisting of two membrane-free compartments: 1) an endothelial cell-lined vascular compartment, and 2) an extracellular matrix-embedded brain tissue compartment (neuroblastoma, astrocytes, and microglia). This plate-based microfluidic platform allows for potentially automated high throughput and high-content imaging with relatively fast readouts. We evaluated four OPs for concentration-dependent effects on: 1) overall cell viability/toxicity within the construct, 2) penetration of OPs across the model BBB, 3) inhibition of AChE activity in target cells following exposure, and 4) residual OP in endothelial vascular compartment.

## Methods

### Chemicals and reagents

Dimethyl methylphosphonate (DMMP, 97%), diethyl methylphophate (DEMP, 97%), diethyl cyanophosphonate (DECP, 90%), and diethyl chlorophosphate (DCP, 97%), (Scheme S1) were purchased from Sigma (St. Louis, MO, USA). Dulbecco’s modified eagle’s medium (DMEM) supplemented with antibiotics for cell culture was purchased from ATCC (Manassas, VA). Fetal bovine serum (FBS) was purchased from ATLANTA biologicals (Flowery Branch, GA). All other reagents were purchased from Sigma and Fisher Scientific unless otherwise noted.

### Cell culture

Complete growth media for all cells (including co-cultures) consisted of DMEM supplemented with 10% FBS and antibiotics. Cryo-preserved bEnd.3 (immortalized murine brain endothelial cells), N2a (immortalized murine brain neuroblastoma), and C8-D1A (immortalized murine astrocytes), were purchased from ATCC. BV-2 (immortalized murine microglia) was obtained from Dr. G. Jean Harry (National Institute of Environmental Health Sciences, Research Triangle Park, NC). bEnd.3, C8-D1A, and BV-2 were maintained as previously described^23^. N2a cells were differentiated prior to incorporation in co-cultures by replacing complete medium with 0.2% FBS in DMEM for one day after passage. The cells were maintained in medium for 2–3 days followed by replacement with FBS-free DMEM one day prior to co-culture.

### 3D brain tetra-culture

The microfluidic chip (OrganoPlate, MIMETAS, the Netherlands) was used for tetra-culture and 3D blood-brain barrier construction. N2a, C8-D1A, and BV-2 cells were harvested and counted in complete growth media. The counted cells for brain compartment were spun down and the tube with the cell pellet was kept cool. The pellet was suspended in the extracellular matrix (ECM); one part 10 × phosphate-buffered saline (PBS) was added to nine parts concentrated rat tail collagen I solution (Corning, Collagen I Rat Tail, 10.21 mg/ml) diluted to 4 mg/ml with DMEM (without supplement), and the pH was adjusted to 7.0–7.4 with 20 mg/ml NaOH. Cells of 2 μl was added to either each gel inlet (brain lane) well of 2-lane. The plate was placed in an incubator (37 °C, 5% CO_2_) for 45 min to allow the gels to polymerize. Harvested bEnd.3 were counted and seeded at 2 μl of 1 × 10^7^ cells/ml into the inlet wells (tunable perfusion lane) of the plate after gelling (brain lane). The plate was put on the side (angle of 75 degrees) for 1 h to allow the bEnd.3 cells to sediment against the gel. After addition of 50 μl medium to the outlet well, the tetra-cells were incubated again on the side (angle of 75 degrees) for 4 h to complete cell attachment. Additional medium (50 μl) was loaded into the inlet well and the plate was placed on an interval rocker (MIMETAS, The Netherlands), allowing bi-directional flow for perfusion. Medium was refreshed every 2 days.

### Fluorescence microscopy

Cells (in 2-lane microfluidic channel) were fixed in 4% paraformaldehyde (PFA) in PBS (phosphate-buffered saline) for 15 min, washed twice with PBS for 5 min, and permeabilized with Triton X-100 (0.1% in PBS) for 5 min. Cells were blocked for 45 min at room temperature with 10% normal donor horse serum in PBS. Subsequently, cells were incubated with primary antibodies for 90 min, washed three times, incubated with secondary antibodies for 60 min and washed three times with PBS. Cells were counterstained with Hoechst (Invitrogen H3570). The following antibodies were used for immunohistochemistry: Claudin-5 (1:50, Invitrogen, 341600), Anti-MAP2 (1:100, Invitrogen PA110005), Goat anti-Rabbit AlexaFluor 568 (1:50, Invitrogen A11036), Goat anti-Chicken AlexaFluor 647 (1:100, Life Tech, A21449). Image data was acquired and processed using two-photon confocal microscope (ZEISS Multiphoton LSM 710) and ZEN software.

### Cell viability

The cell viability was performed by using Hoechst to count all cells (blue), and Ethidium Homodimer (EthD-1, the “dead” agent) to stain the dead cells (red). The cells were washed two times with PBS buffer and incubated for 30 min with concentration of Hoechest (1:2000) and 4 μM EthD-1 in D-PBS. We took fluorescent images, automatically counted cells in image-Pro Plus (Media Cybernetics, Rockville, MD). % dead cells were calculated: % dead cells = (number of red foci/number of blue foci) × 100%^23^.

### OP exposure and residual measurement

Following 5 days of 3D tetra-culture, the medium was removed. Four different OPs (DMMP, DEMP, DECP, and DCP) were dissolved in medium. OPs at concentrations of 10^−1^ to 10^−7^ M were added to the each inlet well of the neurovascular endothelial lane (blood lane). After 24 h, residual concentration of OPs in the blood lane was determined by LC-MS/MS, triple quadrupole mass spectrometer coupled with Shimadzu Prominence HPLC (API 3200, AB Sciex).

### Acetylcholinesterase (AChE) assay

After four different OPs exposure for 24 h, media in brain on chip were taken and stored at −20 °C until AChE assay testing. AChE activities were measured according to the manufacturer’s instruction (Molecular Probes™ Amplex™ Acetylcholine/Acetlycholinesterase Assay Kit), their activity was measured in 200 µl total volume, using a CLARIOstar microplate reader at 25 °C (BMG LABTECH).

## Results

### Dynamic 3D tetra-culture platform

**Figure 1:**
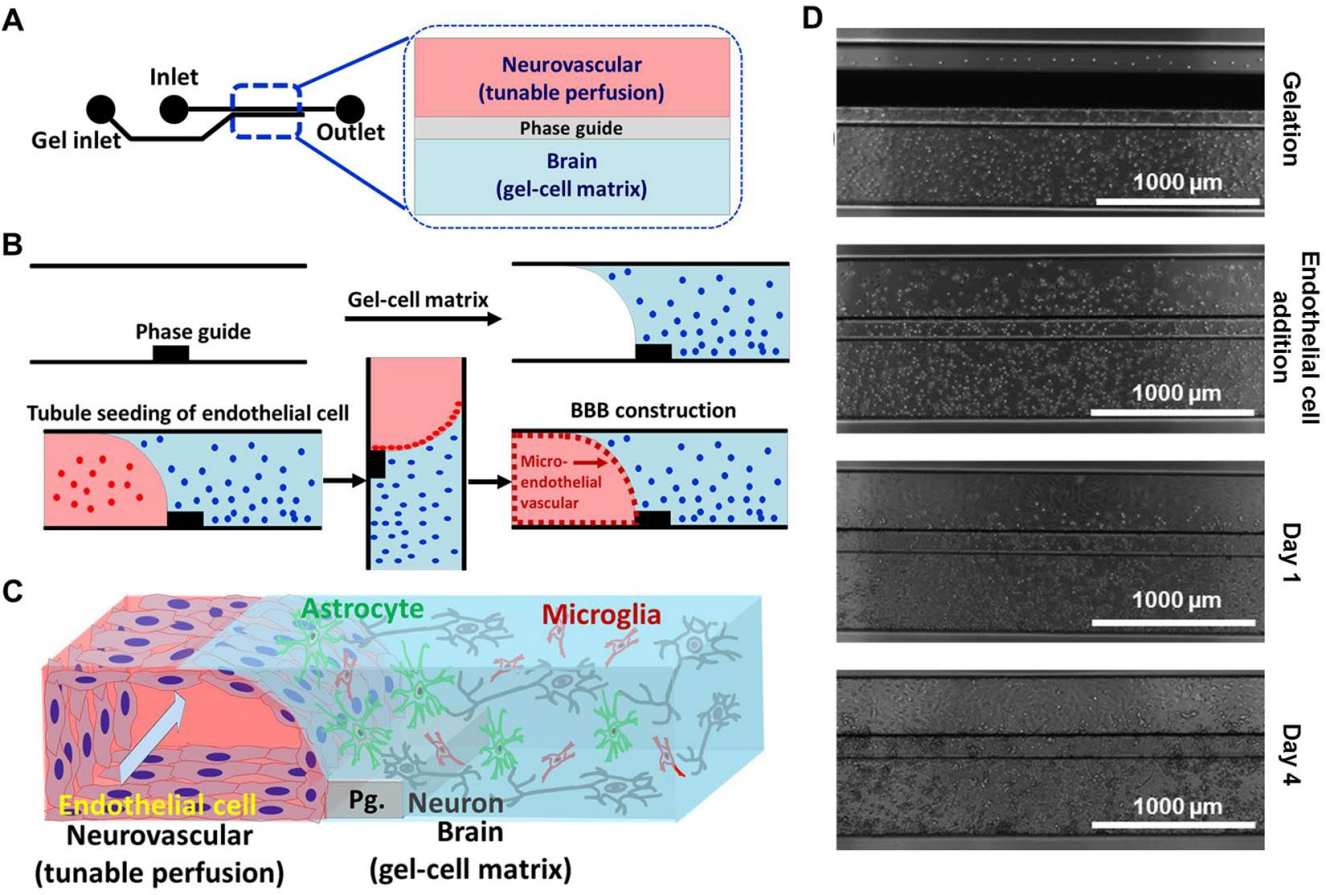
3D-brain construction for high content OPs toxicity screening; (**A**) structure and format application of *in vitro* cultureware, OrganoPlate consisting of 384 well microliter plate with 2 lanes, (**B**) experimental procedure for constructing 3D-neurovascular/brain compartment, (**C**) scheme of 3D tetra-culture for brain on chip, and (**D**) time lapsed images for 3D tetra-culture.

Figure 1 shows the process used to construct the brain on chip. We first loaded collagen-embedded neuroblastoma, astrocyte, and microglia (gel-cell matrix) into the brain lane using capillary pressure barriers called phase guides which enable separation of gel and fluid phases and form a membrane-free substrate for endothelial cell attachment^22,24^ and allowed the gels to polymerize (Fig. 1B). Endothelial cells were seeded against the gel-cell matrix of brain lane. Following stable attachment on the gel-cell matrix, endothelial cells formed a confluent monolayer, followed by formation of a tube-like structure (Fig. 1C). The plate was kept on a perfusion rocker which provided shear stress and fluidic flow. Figure 1C shows final brain tissue construct in which this scheme was successfully implemented for OPs screening. The four different cells grew well in two different compartments while forming a 3D structure (Fig. 1D).

### Integration of brain on chip construct

**Figure 2:**
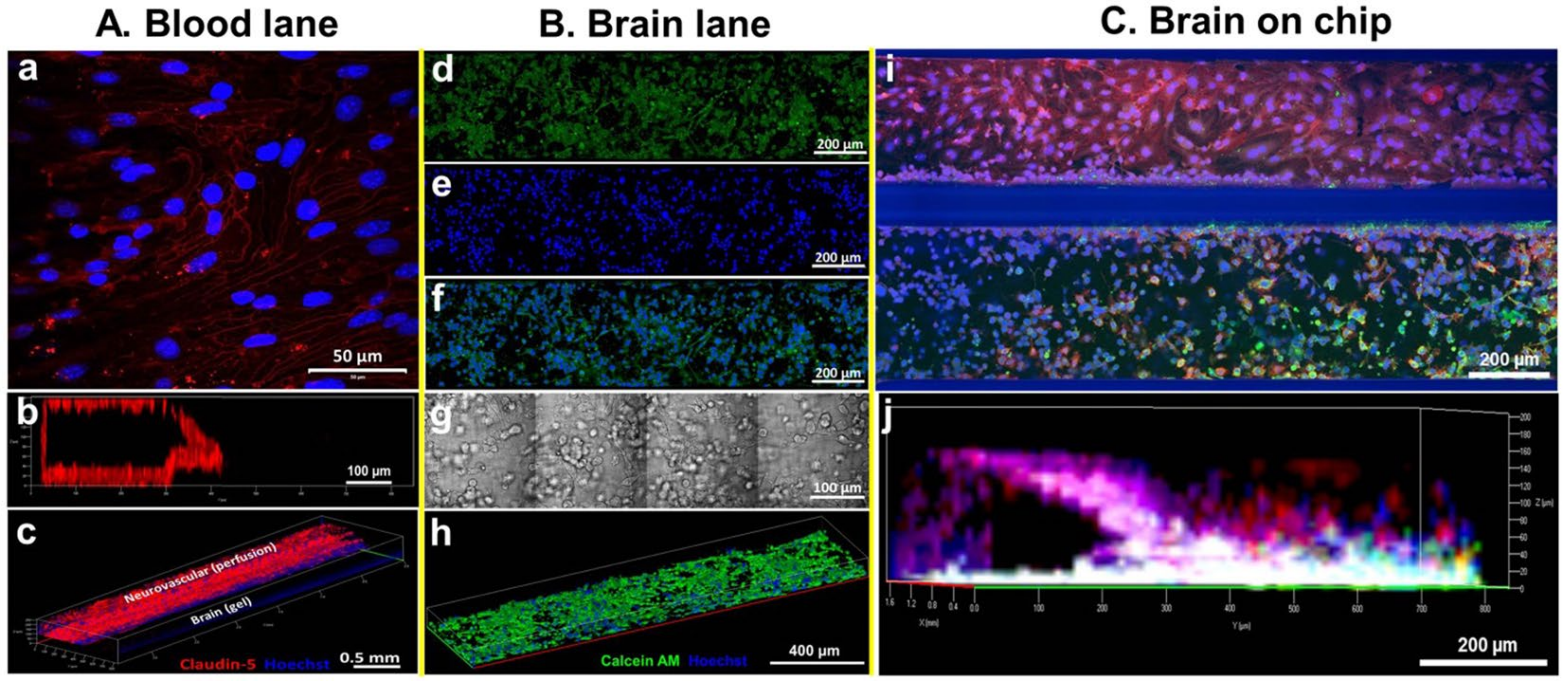
Construction confocal imaging of blood lane and brain lane; (**A**) blood lane only; (a) 2D stained image of tight junction (claudin-5, red) and nucleus (Hoechst, blue) for neurovascular, (b) cross-sectional 3D image of neurovascular, (c) 3D image of neurovascular, (**B**) brain lane only; (d) live (Calcein AM, green), (e) nucleus (Hoechst, blue), (f) merged image of live and nucleus images, (g) bright field image, (h) 3D image of merged construct of gel-3 different type cells matrix, (**C**) Brain on chip by the tetra-culture (claudin-5, red; calcein AM, green, and Hoechst, blue); (i) 2D stained image for blood and brain lanes, (j) 3D images of brain construct with endothelial, neuroblastoma, astrocyte, and microglia cells.

We optimized brain on chip construction by culturing two compartment lanes separately. We first injected collagen matrix containing neuroblastoma and glia into brain compartment and then seeded endothelial cells, 1.0 × 10^7^ cells/ml against to collagen matrix (angle of 75 degrees) in the incubator for 4–6 hours. Following stable attachment of the endothelial cell on the collagen matrix, we placed brain on chip on the programmable rocker which can provide perfusion flow with an average medium flow of 1.5 μL h^−1^ ^25^. As shown in Fig. 2A, endothelial cells formed a confluent monolayer (Fig. 2a), followed by formation of a tube like structure (Fig. 2b,c). Confocal images show neurovascular construct such as tight junction formation (claudin-5 red and nucleus blue).

The brain compartment lane was also optimized by balancing cell density, 2.5 × 10^4^ total cells in collagen gel, gelation time, medium compatibility, and ratio between different cells, astrocyte, neuroblastoma and microglia was 40%: 40%: 20%. Figure 2B shows stained images of brain construct of astrocyte, neuroblastoma and microglia with Calcein AM (green) and Hoechst (blue) after culturing for 5 days. In order to make sure of neuroblastoma differentiation, we cultured neuroblastoma only in collagen matrix in brain compartment lane in serum free medium for one day and we are able to observe neurite growths (Fig. S1). In brain lane (Fig. 2B), the result of three different cells growth can make sure well with cell’s shapes from bright field image (Fig. 2g).

Figure 2C shows confocal images of final brain on chip construct consisting of blood compartment lane (neurovascular with endothelial cell, b.End3 cells) and brain compartment lane (differentiated neuroblastoma N2a, astrocyte C8D1A, microglia BV2) unit. Claudin-5 (red) was stained for endothelial cells, live (green) and nucleus (blue). Figure 2j show confocal image of 3D constructed neurovascular structure with brain. Non-specific binding of claudin-5 (red) in the gel-cell matrix was observed (Fig. 2i and Fig. S2). We tested reproducibility of brain on chip platform. We took one column of 96-well plate of brain on chip and constructed 16 brains. Each brain on chip has cells ranging from 350 to 400 at certain layer in 3D brain construction (Fig. S3).

### OP effects on AChE activity, viability, and residual Ops

**Figure 3:**
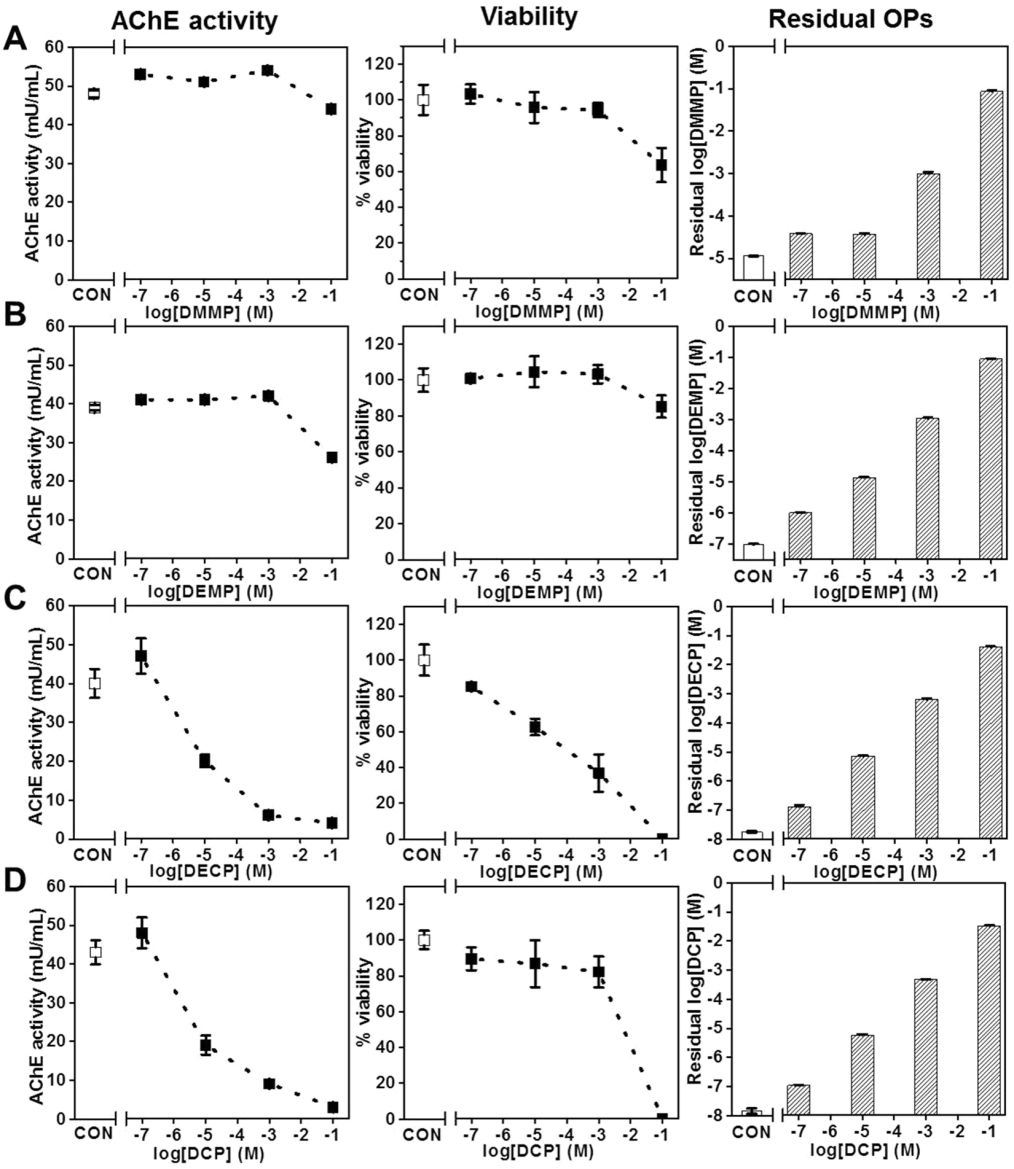
OP exposure results with optimized 3D dynamic tetra-culture brain on chip model. Controls (no OP) are presented on the left side of the broken x axis (labeled CON). AChE activity, viability, and LC-MS/MS data are mean ± S.D., n = 3 technical replicates.

Four OPs were assessed for effects on AChE activity, cell viability, and residual OP in the blood lane using 3D dynamic tetra-culture model. To minimize cross-over of OPs, we added OPs from low concentration to high concentration with 3 replicates (Fig. S3). High-content image analyses were performed for viability and medium samples in blood lane were harvested for AChE analysis and residual OP measurement. These results were compared with control (no OP exposure) across all experiment (Fig. 3). In this model, baselines of control AChE expression/activity and viability were very close across all experiments. We applied concentrations of four OPs ranged from 10^−7^ M to 10^−1^ M for 24 hours. Figure 3 shows the measurement of AChE activity (left column), viability (center column), and residual OPs (right column). Based on AChE inhibition and viability, the results show that DMMP and DEMP (Fig. 3A and Fig. 3B) only have toxic effects on tetra-cultured brain at high concentrations (>10^−3^ M). The results for DECP and DCP show more potent toxic effect on brain tetra-cultured model in terms of AChE inhibition and viability. Concentration-dependent decreases in AChE were observed with DECP and DCP, with estimated IC50’s on the order of 10^−5^ M The residual concentrations of all OPs in the brain compartment correspond to the applied concentrations indicating high permeability across the BBB and metabolic stability within the time frame of the experiments.

### Comparison to *in vivo* data

**Figure 4:**
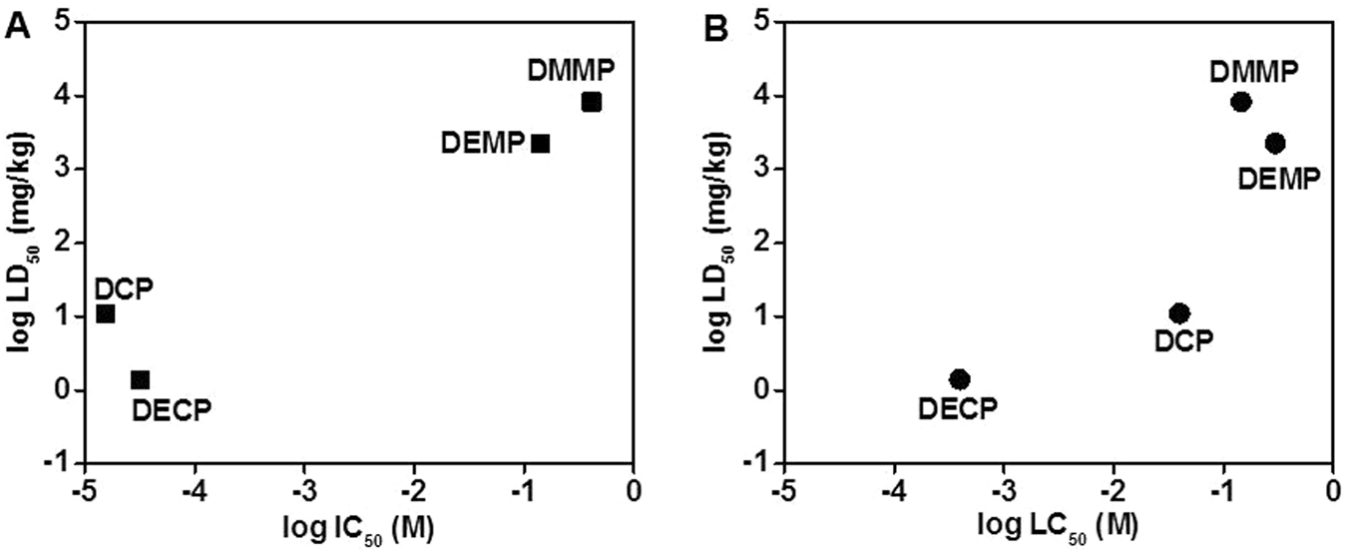
*In vitro* to *in vivo* toxicity correlation based on the log estimated IC_50_ (**A**, for AChE activity), LC_50_ (**B**, for *in vitro* viability), and LD_50_ for DMMP, DEMP, DECP, and DCP: (A) log LD_50_ *vs*. log IC_50_, and (B) log LD_50_ *vs*. log LC_50_.

The compounds tested in this study are mainly used as chemical surrogates for nerve agents, and thus there are relatively few biological data sets available^26–29^. Estimated IC_50_ (AChE activity) and LC_50_ (viability) from Fig. 3 were summarized with LD_50_ *in vivo* for the OPs used in this study (Table 1). Plots between the known *in vivo* data and IC_50_ or LC_50_ showed reasonably good correlations (Fig. 4). From *in vitro* brain on chip model and *in vivo* data in literature, DECP and DCP shows more neurotoxicity than other OPs.

**Table 1:** Comparison of 3D dynamic brain on chip culture model with *in vivo* data^26–29^.

Organophosphates	AChE activity (IC_50_, M)	Toxicity (LC_50_, M)	*In vivo* (LD_50_, mg/kg)
Dimethyl methylphosphonate (DMMP)	4.1 × 10^−1^	1.5 × 10^−1^	8210
Diethyl methylphosphonate (DEMP)	1.4 × 10^−1^	3.0 × 10^−1^	2240
Diethyl cyanophosphonate (DECP)	3.2 × 10^−5^	4.0 × 10^−4^	1.4
Diethyl chlorophosphate (DCP)	1.6 × 10^−5^	4.0 × 10^−2^	11

## Discussion

This platform provides an *in vitro* model of the BBB (endothelial cells and astrocytes) together with cells that mediate brain injury responses (microglia) and surrogates for the primary targets of OP toxicity (neurons) in 3D conditions with dynamic flow and membrane-free co-culture in a 96-well plate format. This represents a unique combination of features enhancing both physiological relevance and potential for throughput not previously achieved with *in vitro* models of the BBB. To demonstrate the utility of this model, we evaluated four OPs for concentration-dependent effects on: 1) overall cell viability/toxicity within the construct, 2) barrier integrity (Fig. S4A), 3) penetration of OP across the model BBB (Fig. S4B), and 4) inhibition of acetylcholinesterase (AChE) activity following exposure.

In this platform, the endothelial layer forms a 3D neurovascular structure against the membrane-free ECM–cells construct (200 μm height), which allows perfusion of nutrition and oxygen to ECM-cells construct and direct cell-cell contact between the parenchymal cells and the endothelium. Even though b.End3 endothelial cells do not form high-resistance tight junctions^24^, we observed significant changes from electrical resistance as well as FITC-Dextran diffusion experiment (Fig. 4S). An advantage of this platform is that the presence of an endothelial barrier creates a more physiologically relevant exposure route than monocultures (or co-cultures) of brain cells being exposed directly to chemicals. While we found that the OPs tested cross the model BBB and rapidly affect AChE activity of neuronal/glial co-cultures, often independent of cell toxicity, the endothelium may serve to reduce the toxicity of these substances by metabolic effects, even if it doesn’t impede their passage into brain tissue. Moreover, the barrier properties of the endothelium play a larger role in determining the neurotoxicity of other chemicals that do not so readily cross the BBB. The exact mechanism of cell death caused by OPs in these constructs is still unknown; however, in most of these experiments cell viability correlated well with reduced AChE activity (Fig. 3). The one exception was DCP (Fig. 3D) where large reductions in AChE activity occurred at lower concentrations than significant reductions in viability, suggesting involvement of additional mechanisms of toxicity such as mitochondrial-mediated and caspase-regulated apoptosis^9–12^.

It is critical to estimate an effective concentration in brain for a given blood concentration for *in vitro* to *in vivo* extrapolation. While physiologically based pharmacokinetic (PBPK) modeling can serve to predict effective concentrations, which eventually predict toxicity based on *in vivo* data, obviously such models really depend on having a priori *in vivo* data. This tetra-cultured brain culture platform has the potential to improve the correlations between *in vivo* data and the *in vitro* model, by providing a more functionally relevant exposure route towards more accurately predicting *in vivo* toxicity.

## Electronic supplementary material

Supporting information
