# Supplementary material for: Three-dimensional (3D) tetra-culture brain on chip platform for organophosphate toxicity screening
Source: Sci Rep. 2018 Feb 12;8:2841. doi: 10.1038/s41598-018-20876-2 (PMC5809488; doi:10.1038/s41598-018-20876-2)
Supplement: Supplementary file 1 — Supporting information [file 41598_2018_20876_MOESM1_ESM.pdf]

Supporting Information

Three-dimensional (3D) tetra-culture brain on chip platform for organophosphate toxicity screening

Youngmi Koo[1,3], Brian T. Hawkins[2,4], and Yeoheung Yun[1*]

[1] FIT BEST Laboratory, Department of Chemical, Biological, and Bio Engineering, North Carolina A&T State University, Greensboro, NC 27411, USA

[2] Discovery Science & Technology, RTI International, Research Triangle Park, NC, 27709, USA

[3] WENeoBio Co., Gunsan-Si, Jeollabuk-do, 54001, South Korea

[4] Duke University Center for WaSH-AID, Durham, NC, 27701, USA

*To whom correspondence should be addressed.

Yeoheung Yun, Ph.D.

North Carolina Agricultural & Technical State University

NSF Engineering Research Center for Revolutionizing Metallic Biomaterials

Fort Interdisciplinary Research Center Building, Room 119

Greensboro, NC 27411, USA

Tel: (336) 285-3226

Fax: (336) 256-1153

E-mail: yyun@ncat.edu

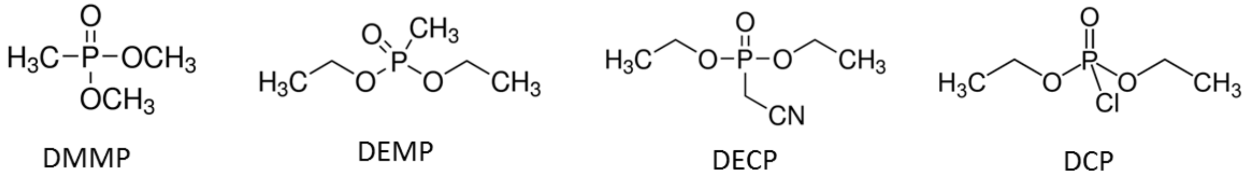

Scheme S1. Chemical structures of organophosphates which used in this article.

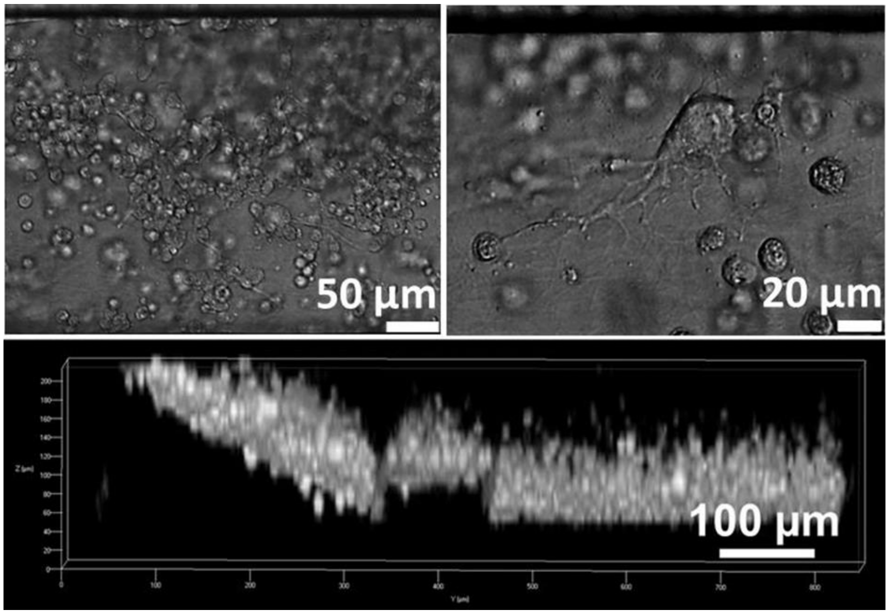

Fig S1. Differentiation of N2a cell in brain lane (top) and 3D cultured structure stained MAP2 with CY5 (bottom).

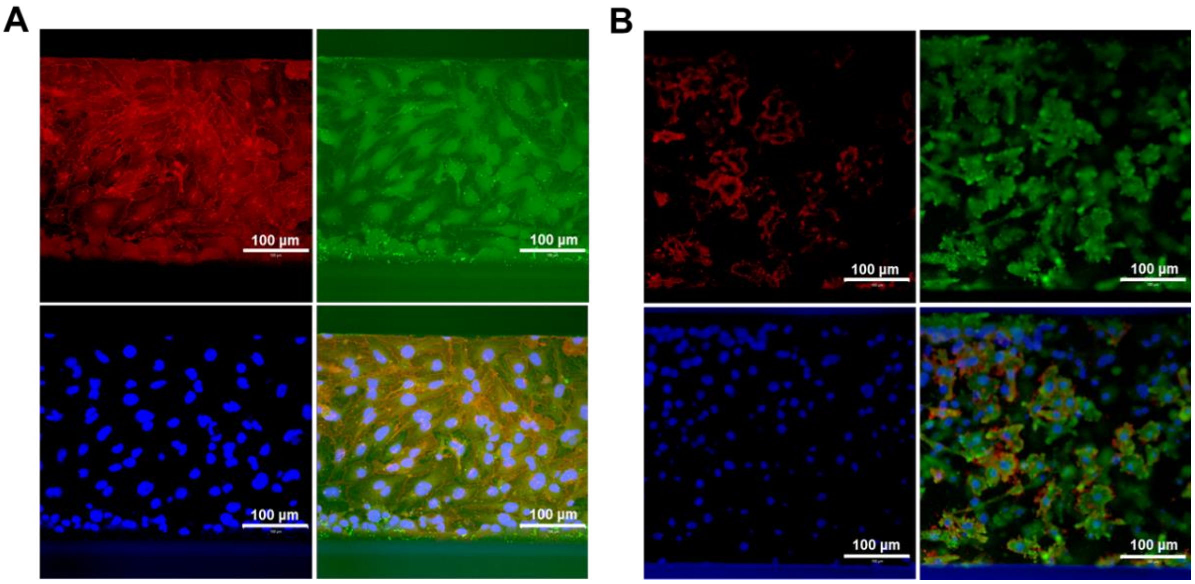

Fig S2. Enlarged images for blood (A) and brain (B) lanes of figure 2C. Red: Claudin-5, Green: Calcein-AM, Blue: Hoechst.

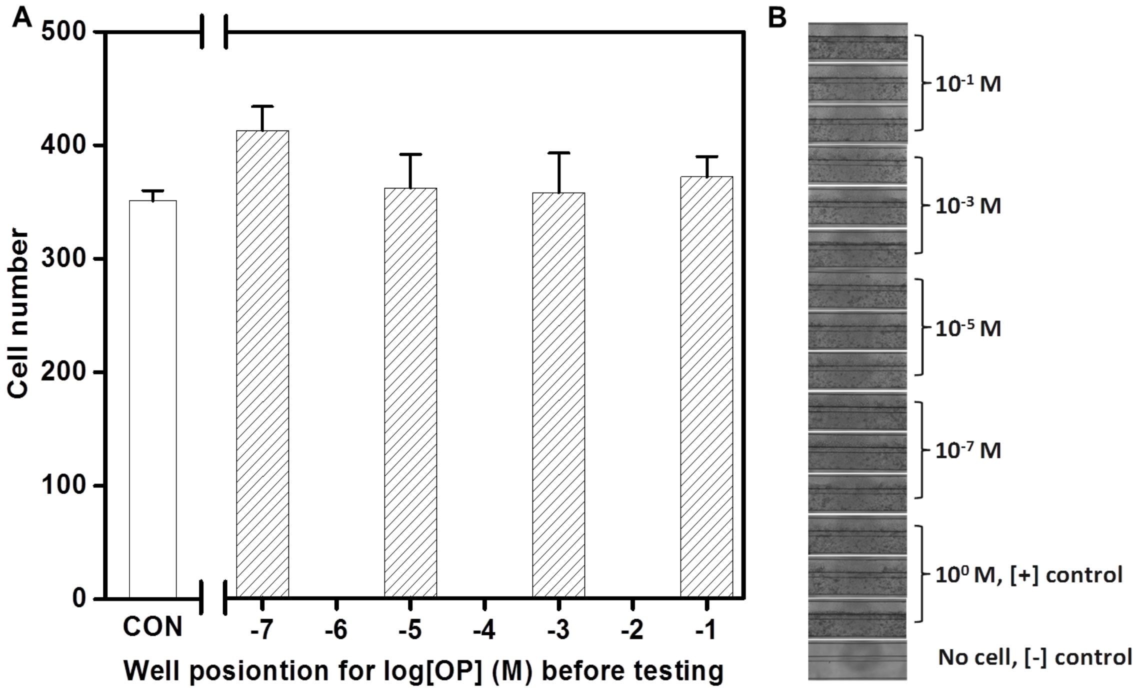

Fig S3. Reproducibility of high throughput tetra-culture method. (A) Cell numbers in the temporary point of 3D structure following the well position, (B) Captured images of the blood and brain lanes.

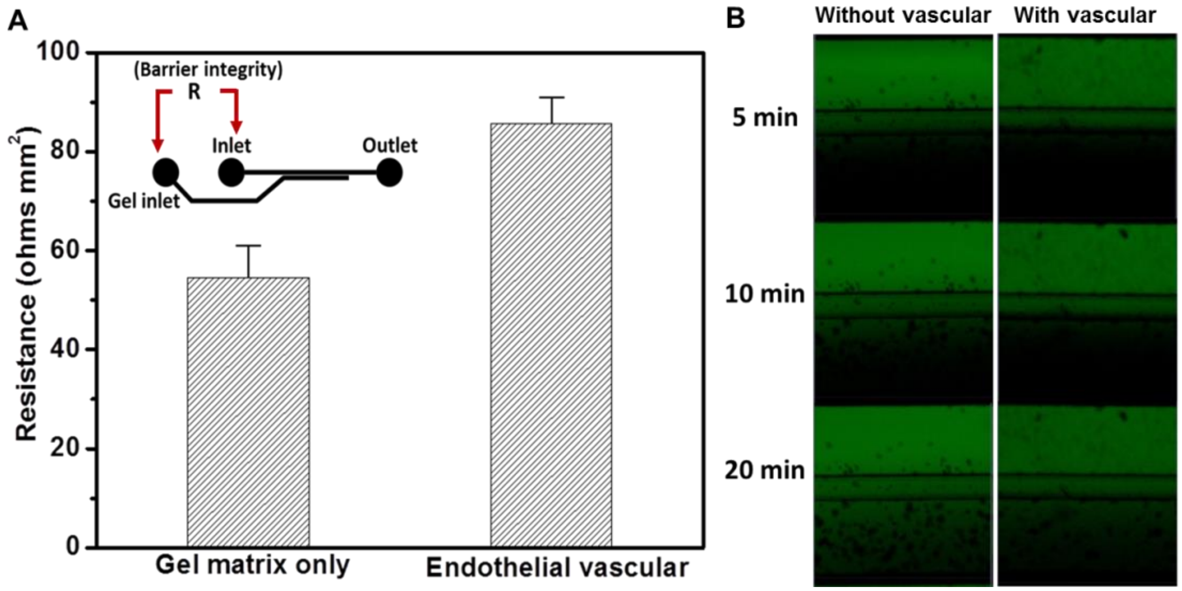

Fig S4. (A) Resistance between brain lane (gel matrix) and blood lane (endothelial vascular) by 4-point measurement, (B) FITC-dextran permeability.

Fig S4-Barrier integrity measurement. Endothelial electrical resistance of confluent b.End3 cells barrier cultured onto brain on chip was measured using an EVOM^2 (World Precision Instruments, Sarasota, FL) with modified electrodes. Chopstick electrode tip was polished by digital electro polishing unit (MultiPrep^{TM} polishin system-8", ALLIED High Tech Products. Inc). As brain on chip is processed to construct such as gelation and endothelialization, electrodes were inserted to the gel inlet well of brain lane and inlet well of blood lane and measure resistance in case of gel matrix only and gel-endothelialization together. Resistance was recalculated by distance between brain lane and blood lane. And endothelial permeability was also determined by adding 10 μM FITC-dextran (MW: 40 kDa) to the blood lane 5 days after seeding endothelial cells on brain on chip. Fluorescent images were acquired as time lapsed images after the addition of the FITC-dextran solution using two-photon confocal microscope

(ZEISS Multiphoton LSM 710 microscope) equipped with motorized stage. Endothelial permeability was estimated from FITC-dextran diffusion, visualized by analyzing fluorescent images.
